# Long-Term Influence of Laser-Processing Parameters on (Super)hydrophobicity Development and Stability of Stainless-Steel Surfaces

**DOI:** 10.3390/ma11112240

**Published:** 2018-11-11

**Authors:** Peter Gregorčič, Marjetka Conradi, Luka Hribar, Matej Hočevar

**Affiliations:** 1 Faculty of Mechanical Engineering, University of Ljubljana, Aškerčeva 6, 1000 Ljubljana, Slovenia; peter.gregorcic@fs.uni-lj.si; 2 Institute of metals and technology, Lepi pot 11, 1000 Ljubljana, Slovenia; marjetka.conradi@imt.si (M.C.); matej.hocevar@imt.si (M.H.)

**Keywords:** laser surface engineering, wetting, superhydrophobic surfaces, laser material processing, surface modification

## Abstract

Controlling the surface wettability represents an important challenge in the field of surface functionalization. Here, the wettability of a stainless-steel surface is modified by 30-ns pulses of a Nd:YAG marking laser (λ = 1064 nm) with peak fluences within the range 3.3–25.1 J cm^−2^. The short- (40 days), intermediate- (100 days) and long-term (1 year) superhydrophilic-to-(super)hydrophobic transition of the laser-textured surfaces exposed to the atmospheric air is examined by evaluating its wettability in the context of the following parameters: (i) pulse fluence; (ii) scan line separation; (iii) focal position and (iv) wetting period due to contact angle measurements. The results show that using solely a short-term evaluation can lead to wrong conclusions and that the faster development of the hydrophobicity immediately after laser texturing usually leads to lower final contact angle and vice versa, the slower this transition is, the more superhydrophobic the surface is expected to become (possibly even with self-cleaning ability). Depending on laser fluence, the laser-textured surfaces can develop stable or unstable hydrophobicity. Stable hydrophobicity is achieved, if the threshold fluence of 12 J cm^−2^ is exceeded. We show that by nanosecond-laser texturing a lotus-leaf-like surface with a contact angle above 150° and roll-off angle below 5° can be achieved.

## 1. Introduction

Inspired by hierarchical surface structures developed by nature [1,2], intensive research efforts employing different methods including chemical etching [3], two-step etching process [4], chemical vapor deposition [5], incorporation of inhibiting agents [6] and laser texturing [7,8] have been invested in the production of similar functionalized surfaces in the laboratory environment in the last decade. Several studies have shown that the surface morphology and chemistry on micro- and nanoscale can be efficiently controlled also by femtosecond [7,8,9,10], picosecond [11] and nanosecond [12,13,14,15,16] laser pulses as well as by continuous wave (CW) lasers [17]. Such laser-induced micro-/nanostructuring leads to a significant improvement of the surface functionality and opens up completely new possibilities in the field of surface engineering [18] for a wide range of applications in photonics, tribology, wettability, heat transfer, and biomedicine [19,20,21,22,23,24].

Laser texturing of different materials, including glasses, semiconductors, polymers and metals enables the production of surfaces with superior wetting properties, which may be exhibited as extreme water repellency [19,25,26], self-healing [27], self-cleaning [28], anti-icing [29], reduced drag in laminar and turbulent flows [30], significantly enhanced heat transfer [31,32], improved corrosion resistance [33,34] and biodegradability [24]. The majority of these studies were made by ultrashort, i.e., pico-/femtosecond lasers. However, in the last three years several authors [12,13,14,15] have shown that similar surface functionalities can be achieved on metal surfaces (without additional coatings) by using more compact and cost-effective nanosecond laser systems that will importantly enhance the widespread use of the developed technology in different fields of application.

In addition to the cost reduction, the real industrial applications also require flexibility, reliability and repeatability of the process [35]. Immediately after laser texturing, the surfaces usually become superhydrophilic with a static contact angle of *θ* = 0°—they are in a saturated Wenzel regime [15]. However, this (super)hydrophilic state is not stable. Consequently, such a textured surface exposed to the air at ambient conditions can develop hydrophobicity with contact angle exceeding 150° and roll-off angle (RoA) below 5° [13,16,25,34]. Under suitable conditions, the final, hydrophobic state becomes stable. Nevertheless, it seems that this development of water repellency depends on a narrow window of different parameters that are still not well understood. Therefore, the main aim of our work is to study the short-term (first two months after texturing) and the long-term (one year or longer) influence of different processing parameters, including fluence, scan line separation, focal position, and wetting during contact angle measurements on the development and stability of superhydrophobic surfaces after nanosecond-laser texturing of low carbon stainless steel in air. The low carbon stainless steel was chosen, since it is widely used in many areas of mechanical engineering and construction industry due to its excellent physical and mechanical properties [36]. Although several studies have been devoted to the examination of how processing parameters influence wettability of metallic surfaces [12,13,14,15,25,33,34,37], all of them were performed only within a short-term period after texturing (2 months or less). Therefore, the important goal of this research is to investigate the wettability development within a much longer period, specifically 1 year after surface texturing. The presented results lead to new important insights into the temporal wettability changes of laser-textured metallic surfaces.

## 2. Materials and Methods

**Materials.** The samples made of commercially available AISI 316L stainless steel had the following chemical composition (in wt. %): Cr 16.9, Ni 10.04, Mo 2.07, Mn 1.84, Si 0.57, Cu 0.41, P 0.036, C 0.019, S 0.0009, V 0.077, Nb 0.015, N 0.044, and Fe the rest. They were laser-textured in the as received state without any polishing. The *S*_a_ roughness of the as received samples equaled 0.175 ± 0.02 μm. Before processing, all samples were ultrasonically cleaned in distilled water for 12 min and rinsed with ethanol.

**Laser texturing.** Surfaces of the AISI 316L samples with dimensions of 20 × 20 × 1 mm^3^ were textured by a marking nanosecond Nd:YAG pulsed laser (LPKF, Ljubljana, Slovenia, OK DP10) radiating pulses with a wavelength of 1064 nm and duration of 30 ns. The laser beam was guided across the surface by a scanning head (Scanlab, Puchheim, Germany, SCANgine 14) equipped with an F-Theta focusing lens (focal length of 160 mm) resulting in a focal beam waist radius of 29 μm. All experiments were done by a constant pulse frequency of 25 kHz and using a constant marking speed of 150 mm/s, resulting in 90% overlap of consecutive spots. Some samples were textured only in the *x* direction (0°), while others were textured in two directions (0°/90°), first in the *x* (0°) and then in the *y* (90°).

**Experiments with different fluences.** To examine the influence of laser fluence on the wettability development, we prepared 64 different samples. Half of them, i.e., 32 (labeled S1–S32), were textured in both directions (0°/90°), while the others (labeled S33–S64) were textured only in *x* (0°) direction. The distance between adjacent laser scanning lines (i.e., the scan line separation, *Δx* and *Δy*) equaled 50 μm for both directions and all samples. In these experiments, the peak fluences were varied within the range 3.3–25.1 J cm^−2^. The detailed processing parameters are listed in Table S1. All the experiments were performed twice to confirm the repeatability of the results.

**Experiments with different micro-morphologies.** The influence of different texturing patterns on the wettability development were examined in 16 samples, labeled S65–S80. Here, we tested the following additional scan line separations: 10 μm, 100 μm, 200 μm. At each scan line separation, two 0°/90° and two 0° samples were textured. Each of these two samples was processed with one of the following peak fluences: *F*_1_ = 12.1 J cm^−2^ and *F*_2_ = 25.1 J cm^−2^. The detailed processing parameters are listed in Table S2.

**Experiments at different focal positions.** To study the influence of focal position, we prepared two samples, labeled S81 and S82 with different laser parameters. In all cases we used the same fluence *F* = 12.1 J cm^−2^ and 0°/90° pattern with scan line separation of *Δx* = *Δy* = 50 μm. Here, sample S81 was processed in the focal position (*Δz* = 0), while sample S82 was moved 600 μm from this position towards the F-Theta lens (*Δz* = −600 μm). Experiments with each S81–S82 sample were repeated 9 times. The processing parameters are collected in Table S3.

**Surface characterization.** The samples morphology was characterized by using a scanning electron microscope (SEM; JEOL JSM-6500F) and by non-contact 3D optical microscopy (Alicona G4 3D optical Infinite-Focus Measuring device).

**Wettability measurements.** Evolution of wettability was analyzed by measuring the static contact angle *θ* using a goniometer of our own design. The goniometer consists of a CCD camera (Basler AG, Ahrensburg, Germany, scA1400-17fm, 1.4 Mpx) equipped with a microscopic objective and micrometer syringe enabling delivery of distilled water droplet with a volume of 5 μL.

When the image of a water droplet applied to the tested surface was captured, we fitted *(i)* the line to the interface between the solid surface and the surrounding air; and *(ii)* the circle to the liquid-gas interface. Here, the control points were manually added in a way that the circle mainly corresponds to the liquid-gas interface in the vicinity of both contact points between all three phases: gas, liquid and solid. The contact angle was estimated as the angle between the fitted circle and the fitted line, as shown by Figure S1 for the symmetrical and asymmetrical droplet.

The short-term wettability development was studied within the first eight weeks after laser texturing. The first measurement was performed immediately after processing and was then repeated within the first two weeks every second day. Within the following two weeks, the wettability was measured every fourth day, while during the last four weeks, it was measured every second week. The long-term wettability of all samples was also examined one year after the texturing. After each measurement, the samples were dried by using a hot air gun (at 150 °C).

For comparison, the wettability of the as-received (unprocessed and unpolished) sample was measured and equaled 95.0 ± 6.4°. The reason of slightly hydrophobic nature of the as-received metallic samples most probably lies in micro/nanoroughness that leads to the Cassie-Baxter regime (that can turn, opposite to the Wenzel regime, hydrophilic material into a hydrophobic state). However, the Young angle of the base material should be measured on ideal (highly polished) surface [38,39]. Therefore, we performed this measurement on polished samples with *S*_a_ = 25 ± 2 nm. This way, the measured Young angle equals *θ*_Y_ = 81.6 ± 5.7°.

**Studying the wetting influence on wettability development.** Additional four samples, labeled S83-S86 were prepared with the same parameters as S81 (e.g., see Table S3) to study the effect of wetting due to static contact angle measurements on the wettability development. The evolution of wettability on these samples was evaluated within 40 days after texturing. Here, wettability of sample S83 was measured immediately after the laser texturing and then every 2nd day. The wettability on sample S84 was firstly measured 4 days after the texturing and then every 4th day, the wettability on sample S85 was firstly measured 8 days after the texturing and then every 8th day, while in the case of sample S86 we firstly measured wettability 16 days after the texturing and then every 16th day.

Again, the samples were dried by using a hot air gun (at 150 °C) after each measurement.

## 3. Results

### 3.1. Surface Morphology after Laser Texturing

**Figure 1:**
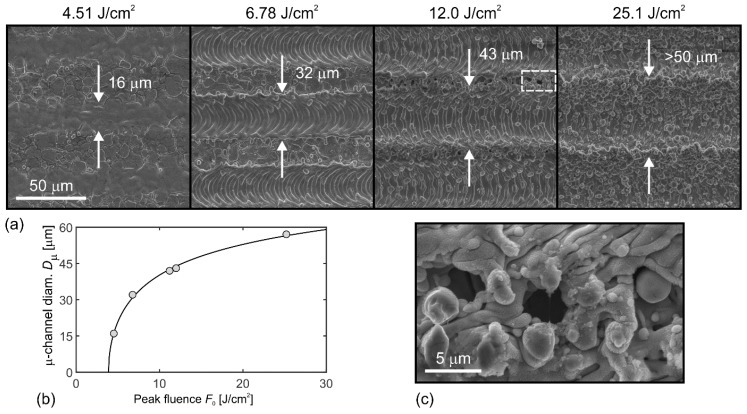
(**a**) SEM micrographs of μ-channels at different peak fluences *F*_0_ (shown on the top of each micrograph); scan line separation in all cases equaled *Δx* = 50 μm. (**b**) Experimental (points) and theoretical (line; see Equation (2)) dependence of μ-channels diameter on peak fluence *F*_0_. (**c**) Magnification of the selected area in (**a**) showing the appearance of μ-cavities.

Overlapping of laser spots leads to the formation of micro(μ)-channels, as clearly visible on SEM micrographs in Figure 1a. Here, μ-channels induced by 0° texturing with scan line separation of *Δx* = 50 μm are shown. It is clearly visible that μ-channel diameter *D*_μ_ (marked by the white arrows) depends on pulse fluence. This dependence can be described by using a Gaussian spatial profile [11], where the fluence *F*(*z*,*r*) as a function of focal position *z* and radius *r* is given by:

(1) $$\;F(z,r)=F_{0}\frac{w_{0}^{2}}{w^{2}(z)}\exp\left(-2r^{2}/w^{2}(z)\right).\;$$

In Equation (1), *F*_0_ stands for the peak fluence at *r* = 0 and *z* = 0, *w*_0_ is the 1/e^2^ beam waist radius at the focal position (at *z* = 0), while *w*(*z*) stands for the beam radius as a function of *z*. If the surface is placed in the focal position, Equation (1) reduces to $F(r)=F_{0}\exp(-2r^{2}/w_{0}^{2}).$ Laser ablation occurs in the region where the fluence *F*(*r*) exceeds the threshold fluence *F*_th_ for ablation. Therefore, the μ-channel diameter *D*_μ_ as a function of peak fluence *F*_0_ can be described as:

(2) $$\;D_{\mu }=w_{0}\sqrt{2\ln\left(\frac{F_{0}}{F_{th}}\right)}.\;$$

It should also be noted that the peak fluence *F*_0_ is twice higher than the average fluence *F* (also called the pulse fluence) normalized to the beam waist radius:

(3) $$\;F_{0}=2F=2\frac{E_{p}}{\pi w_{0}^{2}},\;$$

where *E*_p_ stands for the pulse energy.

From the SEM micrographs (see Figure 1a and Table S4) we measured the μ-channel diameters. In the case of the highest peak fluence *F*_0_ (the last micrograph in Figure 1a), the *D*_μ_ cannot be determined, since it exceeds scan line separation, *Δx* = 50 μm.

The measured diameters *D*_μ_ as a function of peak fluence *F*_0_ are shown as dots in graph in Figure 1b. Here, the solid line presents the fit of Equation (2) to the measured data by using least-square method. From this fit we obtained the fluence threshold as *F*_th_ = 3.8 J cm^−2^. The fit further enabled us to estimate the μ-channel diameter at *F*_0_ = 25.1 J cm^−2^; it equals *D*_μ_ = 56 μm > *∆x* and, consequently, results in overlapping of the adjacent μ-channels. This calculated result is experimentally confirmed by μ-channel diameter measurements at the same fluence but for *∆x* = 100 μm (see Table S4).

Since the surface topography significantly depends on the combination of peak fluence and scan line separation, the variation of these two parameters can be used for structuring different surface topographies. In a special case, when *D*_μ_ is just slightly smaller than scan line separation *∆x*, the μ-cavities appear on the border between two adjacent μ-channels as clearly visible from Figure 1c that is the magnification of the selected area (marked by the white rectangle) in the third micrograph of Figure 1a. Such μ-cavities play an important role in engineering of surfaces for enhanced heat transfer, as explained, demonstrated and proved in Refs. [31,32].

**Figure 2:**
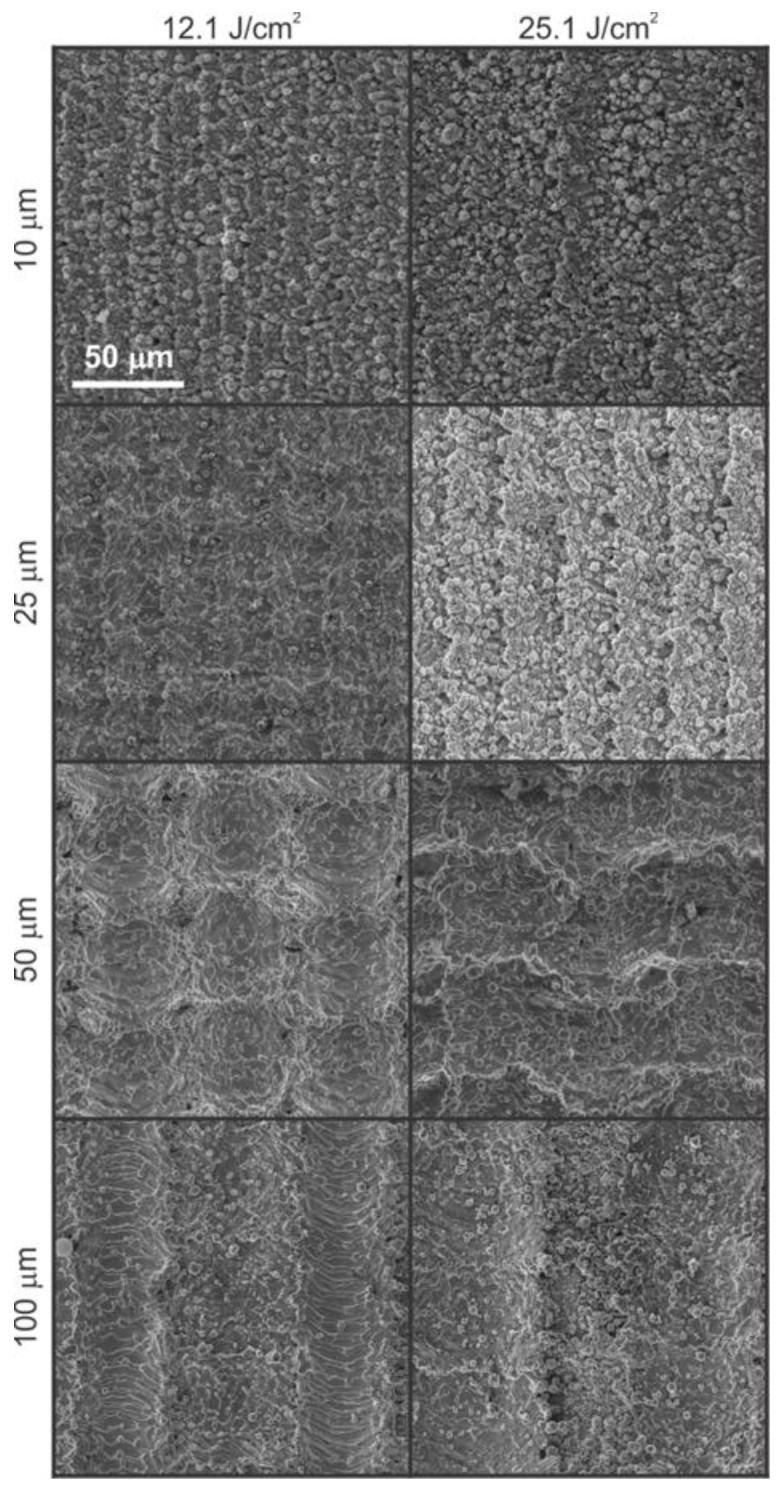
SEM micrographs of surfaces processed by two different fluences F_0_ (shown on the top of each column) and with different scan line separations *Δx* = *Δy* (shown on the left-hand side of each row).

The importance of the scan line separation is shown in Figure 2. Here, 0°/90° texturing with different scan line separations *∆x* = *∆y* was used at two different fluences. The surface was first textured in *x* (0°) and then in *y* (90°) direction. As it has already been shown [34], the second beam pass is more pronounced. From Figure 2 it can be seen that decreasing scan line separation and/or increasing (peak) fluence leads to higher surface porosity. This is caused by to the increased overlap between the adjacent μ-channels. The high-magnification SEM images are presented in Figures S2–S4.

For surfaces in Figure 2, we also measured the average surface roughness *S*_a_ by using 3D microscopy. The results, listed in Table 1, indicate that the lowest value of *S*_a_ is measured for *∆x* = *∆y* = 10 μm for both fluences due to the highest surface porosity of the two surfaces. A further increase in *S*_a_ is observed with increased scan line separation until *D*_μ_ is smaller than *∆x* = *∆y*, reaching a peak value for both fluences at *∆x* = *∆y* = 50 μm. After this point, the additional increase of scan line separation results in decreased average surface roughness due to the appearance of an unprocessed area between two adjacent, well-separated μ-channels. Similar dependence on surface roughness as a function of scanning line separation was shown by Conradi et al. [20].

**Figure 3:**
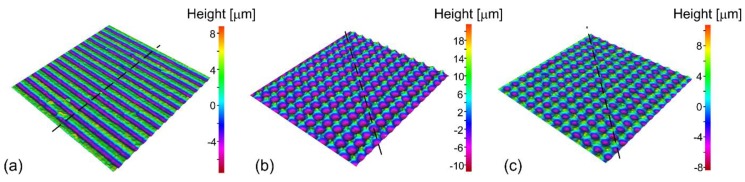
3D profile of (**a**) 0° textured surface at focal position, (**b**) 0°/90° textured surface at focal position and (**c**) 0°/90° textured surface out of focal position at *z* = −600 μm (towards the laser). All surfaces are processed at *F*_0_ = 12.1 J cm^−2^ with *Δx* = *Δy* = 50 μm.

Typical 3D surface topographies are shown in Figure 3. When 0° texturing is applied, the lines appear on surfaces as visible in Figure 3a. On the other hand, the comparison between 0°/90° texturing in the focal and out of the focal position (at *z* = −600 μm, i.e., 600 μm towards the focal lens) is revealed in Figure 3b,c, respectively. It can be seen that in this case, the μ-channels are opened in the last scanning direction (90°) as has been already been shown by Trdan et al. [34]. Here, the μ-holes appear where both texturing directions cross each other.

**Figure 4:**
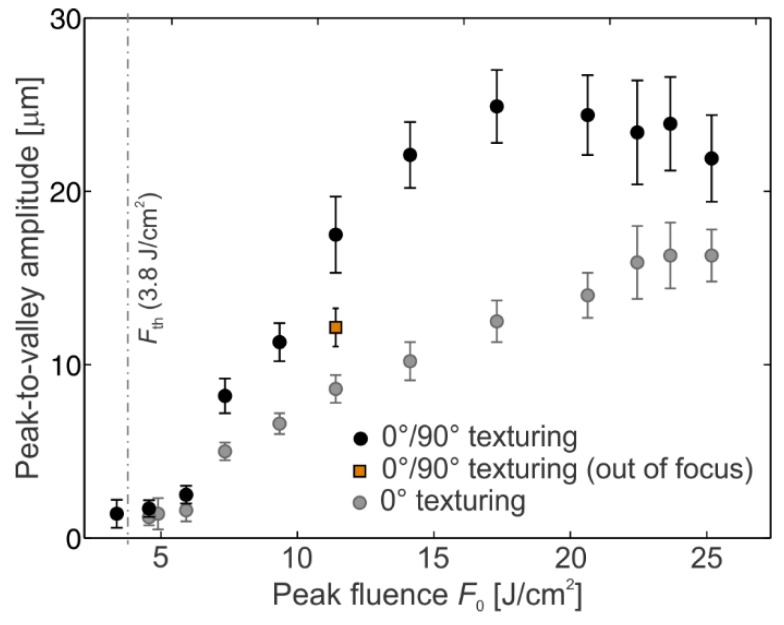
The average peak-to-valley amplitude as a function of the peak fluence *F*_0_. The dependence for 0°/90° texturing in a focal position (black dots), 0° texturing in a focal position (gray dots) and 0°/90° texturing out of the focal position at *z =* −600 μm at *F*_0_ = 12.1 J cm^−2^ (the orange square) are shown.

From the 3D profiles we measured the peak-to-valley amplitudes (PVA) as a function of peak fluence *F*_0_, when surface is textured with scan line separation *Δx* = *Δy* = 50 μm. The obtained dependence is presented in Figure 4. Here, the PVA value was obtained as average peak-to-valley amplitude across the black dashed lines in Figure 3. The black dots in Figure 4 represent average PVA for 0°/90° texturing in focal position, while the gray dots stand for average PVA for 0° texturing in the focal position. The average PVA at *F*_0_ = 12.1 J cm^−2^ when the sample is placed 600 μm towards the focusing lens is presented for the reference in Figure 4 as the orange square. The error bars in Figure 4 show standard deviation.

It is clearly visible that PVA increases approximately linearly for peak fluences *F*_0_ between the threshold value *F*_th_ and the saturated value *F*_sat_. In the case of 0° texturing, the PVA increases for factor of *k*_0°_ = 0.81 μm/(J cm^−2^) and the saturated value PVA_sat_ ≈ 16 μm is achieved at *F*_sat_ = 22.6 J cm^−2^. On the other hand, the linear coefficient *k*_0°/90°_ = 1.92 μm/(J cm^−2^) is twice higher for 0°/90° texturing. In this case, the saturated value PVA_sat_ ≈ 23 μm is obtained at *F*_sat_ = 15.6 J cm^−2^. In case of processing out of the focus, the PVA value is between the values for 0°and 0°/90° texturing in a focal position (for the same pulse fluence).

### 3.2. Influence of Pulse Fluence on Surface Wetting Properties

The wettability was evaluated by measuring the static contact angle. Although some authors [40] denounce the static contact angle analysis since this angle can reach any value within the range of angles between the advancing and the receding contact angle, we made such an analysis to compare our results with previous experiments [12,13,14,15,25,33,34,37] that used similar methodology. However, we also list the roll-off angle (RoA) where applicable.

**Figure 5:**
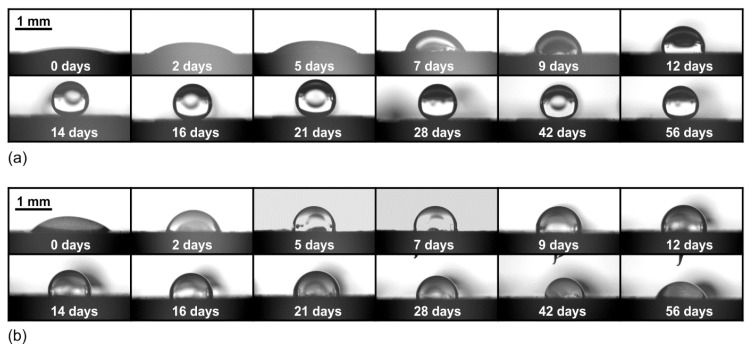
Short-term development of (**a**) hydrophobicity with a stable contact angle (at *F*_0_ = 12.1 J cm^−2^) and (**b**) hydrophobicity with an unstable contact angle (at *F*_0_ = 5.5 J cm^−2^).

**Figure 6:**
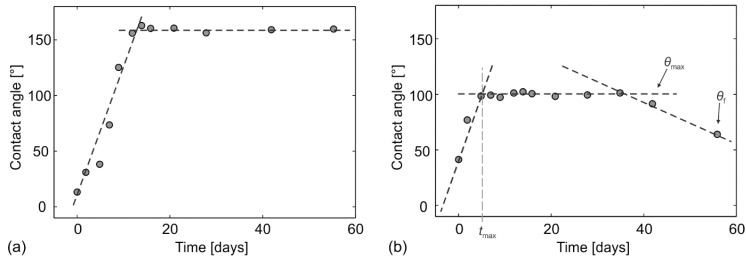
Short-term contact angle development for (**a**) surface exhibiting hydrophobicity with stable contact angle, processed at *F*_0_ = 12.1 J cm^−2^ and (**b**) surface exhibiting hydrophobicity with unstable contact angle, processed at *F*_0_ = 5.5 J cm^−2^. Both surfaces were processed with *Δx* = *Δy* = 50 μm.

The short-term development of the static water contact angle we examined on surfaces that were processed with a scan line separation of *Δx* = *Δy* = 50 μm at two different fluences, 12.1 J cm^−2^ and 5.5 J cm^−2^ (Figure 5). As evident from the temporal contact angle development (Figure 6a), the (super)hydrophobicity on the surface processed at 12.1 J cm^−2^ is developed gradually, with linear increase of the static water contact angle (*κ* = *θ*_max_/*t*_max_ ≈ 11 deg per day) in the first 13 days until stable water-repellency was achieved. After this time, the surface then remains superhydrophobic with a stable contact angle of *θ*_max_ = *θ*_f_ ≈ 159° ± 2° and a RoA of 4.3° ± 0.50. We have measured the wettability of the same sample again after one year (i.e., so called long-term measurement) and the contact angle equaled to 157° ± 3°, while the RoA was still below 5°. This proves the long-term stability of these samples.

On the contrary, the surface processed with a lower peak fluence, 5.5 J cm^−2^, does not develop a stable contact angle in the short-term (2 month) period (Figure 6b). At the beginning, its behavior is similar to the surface textured at a higher fluence, but the contact angle increases more rapidly (*κ* = *θ*_max_/*t*_max_ ≈ 20 deg per day). After the first 5 days, the surface saturates in the hydrophobic limit by achieving the contact angle *θ*_max_ = 100°, with no RoA since the water droplet remains stuck to the surface even when inclined for 90°. The surface remains (stably) hydrophobic for approximately 30 days; after this time, the contact angle starts to decrease and after two months it reaches the “final” contact angle of *θ*_f_ = 65° (with no RoA). However, the long-term measurement after one year revealed that stable *θ*_f_ equals 91° (with no RoA), which is close to the static contact angle of the non-processed sample.

To evaluate and compare the wettability development after laser texturing with different parameters, we propose to use the following characteristics as wettability metrics (see also Figure 6b):

- the maximal contact angle, *θ*_max_ which is defined as a static (apparent) contact angle achieved within the measured (short- or long-term) period;
- the time, *t*_max_, defined as the time in which the maximal contact angle, *θ*_max_, is achieved;
- and the final contact angle *θ*_f_, defined as the apparent contact angle, measured at the end of the evaluating period—as clearly demonstrated by Figure 6b, this is a very vague parameter/metric especially in short-term measurements that are presented and discussed by the majority of the published papers [12,13,14,15,25,33,34,37] reporting on the laser-induced wettability control.

**Figure 7:**
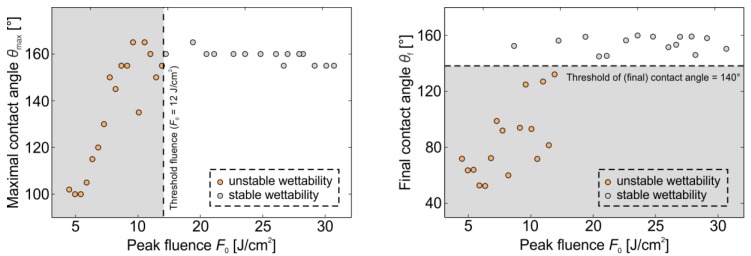
(**a**) Maximal contact angles and (**b**) final contact angles as a function of the peak fluence *F*_0_. All results are measured for *Δx* = *Δy* = 50 μm.

The maximal and the “final” contact angles of surfaces, processed with different laser fluences (for *Δx* = *Δy* = 50 μm) were evaluated as a function of pulse fluence. The results are presented in Figure 7. Here, the results of unstable wettability (similar behavior as in Figure 6b) are marked by the orange circles, while the stable superhydrophobic surfaces (e.g., with behavior similar to that in Figure 6a) are shown with the gray circles. Figure 7a shows the maximal contact angle as a function of fluence and indicates the threshold peak fluence of 12 J cm^−2^ for achieving stable wettability. This threshold fluence was determined as an average value of fluences used to process the last unstable sample and the first stable sample. In this case, the threshold final contact angle of 140° is exceeded, as visible from Figure 7b showing the final contact angle as a function of the peak fluence.

### 3.3. Influence of Scan Line Separation on Surface Wetting Properties

We have also analyzed the short-term and the long-term wettability development for different scan line separations (0° texturing), *Δx* = 10 μm, 100 μm, 200 μm and for the net-textured surfaces (0°/90°) *Δx* = *Δy* = 10 μm, 100 μm, 200 μm. All the samples were processed at the same fluence of 25.1 J cm^−2^ (significantly exceeding the threshold fluence for the development of the stable superhydrophobicity—see Figure 7a).

**Figure 8:**
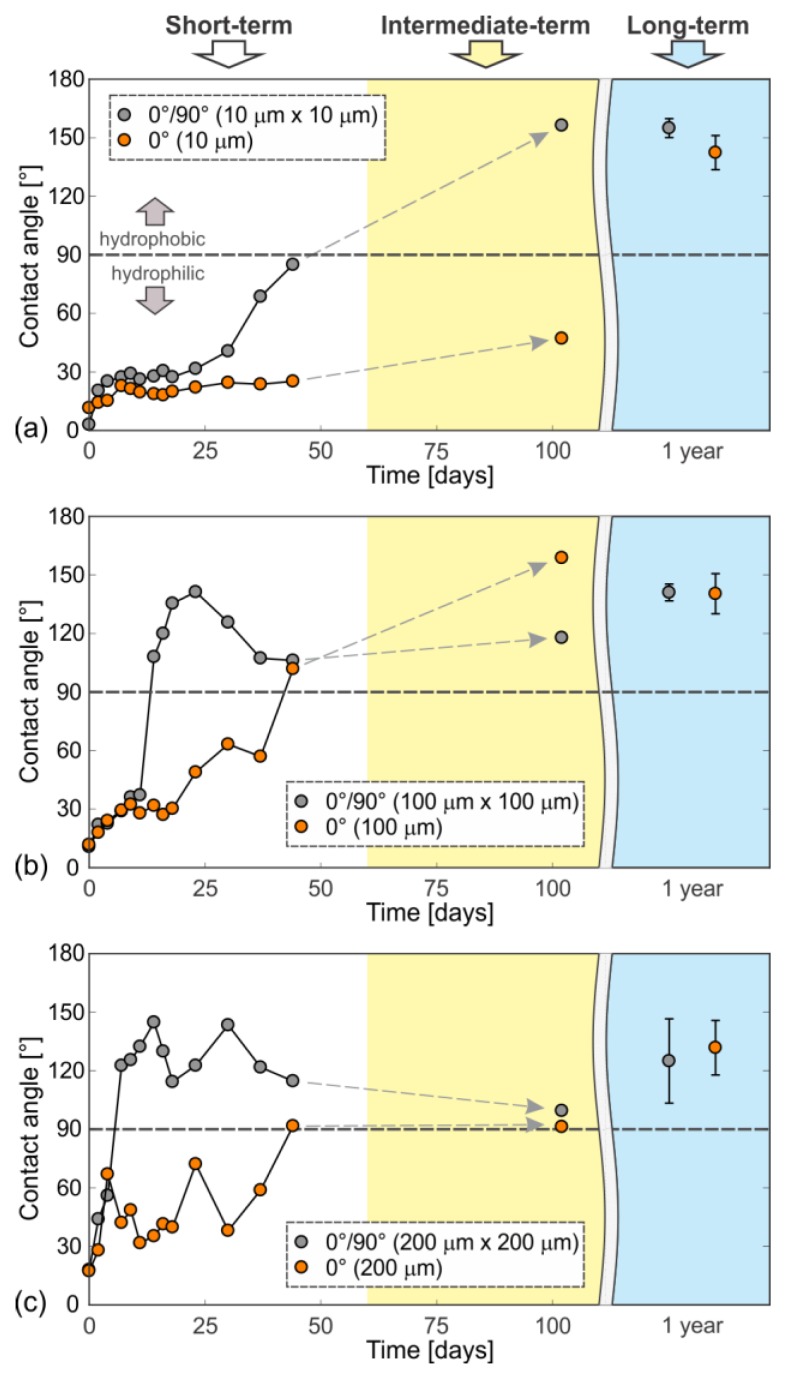
Short-term, intermediate-term and long-term static water contact angle development on surfaces with the following scan line separations: (**a**) *Δx* = *Δy* = 10 μm; (**b**) 100 μm; and (**c**) and 200 μm.

Short-term measurements include a 40-day contact angle evaluation; the wettability was measured again after 100 days (intermediate-term measurement), while the long-term measurements of the contact angle were performed after 1 year. The results are presented in Figure 8.

The samples with *∆x* = 100 μm and *Δx* = *Δy* = 10 μm turned highly hydrophobic with contact angles of around 141°. The samples with scan-line separations of *∆x* = 200 μm and *Δx* = *Δy* = 200 μm remained moderately hydrophobic with contact angles between 125° and 132°. However, the superhydrophobicity as defined by Wang and Jiang [41] was—in this case—achieved only for the surfaces with smaller scan-line separations, (e.g., *Δx* = *Δy* = 50 μm—Figure 7; and *Δx* = *Δy* = 10 μm—Figure 8a). Here, the measured contact angles were up to 159° (>150°), while the measured RoA equaled 3.0° ± 0.5°. That means that if this surface is tilted for more than 3°, the water droplet roll off the surface and cleans the dust pieces put on the surfaces—it expresses the self-cleaning effect [42] (see also Figure S5).

### 3.4. Influence of Focal Position on Surface Wetting Properties

**Figure 9:**
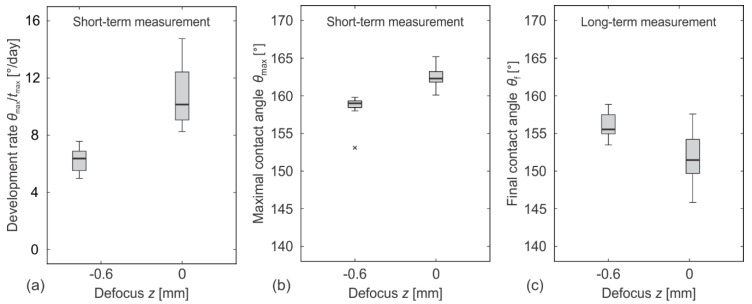
The wettability parameters as a function of the focal position *z*. (**a**) The average development rate, measured within 2 months; (**b**) the maximal contact angle within 2 months; and (**c**) the final contact angle after 1 year. The sample were processed by *F*_0_ = 12.1 J cm^−2^ and *Δx* = *Δy* = 50 μm.

Some recent results [14] show that wettability gradients can be achieved by processing a metallic sample at different focal positions. Therefore, we have examined the short-term and the long-term wettability of the laser-textured surfaces as a function of the focal position. The results for the surfaces processed in the focal position (i.e., at *z* = 0) and at *z* = −600 μm (that in our case equals to 22% of the Rayleigh length, *z*_R_ = 2.7 mm) are presented in Figure 9.

Figure 9a shows the development rate *θ*_max_/*t*_max_, i.e., the average increase in a contact angle per day from the superhydrophilic state (the saturated Wenzel regime, with *θ* = 0°) to the maximal contact angle *θ*_max_, measured within 2 months (the so-called short-term period). This indicates that the surface processed out of the focus (the left box in Figure 9a) expresses a more hydrophilic behavior and develops hydrophobicity slower than the sample processed in the focal position. However, such wettability differences are not stable by time, since the superhydrophobic state was achieved by both surfaces within a short-term period (2 months after the processing) as can be easily seen from the box plots in Figure 9b presenting the maximal contact angles of the surfaces, processed in and out of the focal position. The wettability of the same surfaces was evaluated again after one year and the results are presented in Figure 9c.

### 3.5. The Effect of Wetting Period on Hydrophobicity Development

**Figure 10:**
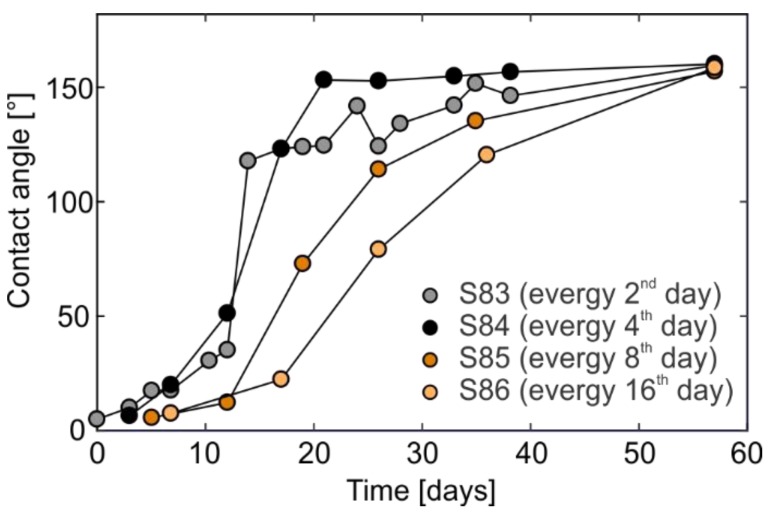
Contact angle development for different wetting periods due to contact angle measurements.

It is impossible to measure the surface wettability without wetting the surface. Therefore, four different surfaces (S83–S86; Table S3) were textured with the same processing parameters *Δx* = *Δy* = 50 μm; *F*_0_ = 12.1 J cm^−2^), but measurements on each of them were conducted at different periods. The first sample (S83) underwent measurements every second day, the S84 every fourth day, the S85 every eighth day, while measurements on the S86 sample were conducted every sixteenth day. The wettability development for all the measured surfaces is shown in Figure 10.

The presented results indicate that the surface wetting also influences the wettability transition, but it has no significant influence on the final results—all the surfaces achieved a similar contact angle of *θ* ≈ 150° and RoA < 5° after approximately 2 months. The wettability of all of these surfaces were examined again after one year and at that time, the similar contact angles and RoAs were measured as after 40 days. 

## 4. Discussion

The results of wettability measurements at different fluences (Figure 5 and Figure 6) indicate that some laser-textured surfaces have the ability to develop stable hydrophobicity, while the others exhibit a decrease of water-repellency after the highest hydrophobicity is achieved. In case of *Δx* = *Δy* = 50 μm stable hydrophobicity is achieved already within the short-term period (e.g., within 2 months), if the peak fluence exceeded the threshold fluence of 12 J cm^−2^ (Figure 7). In this case, the final contact angle (at stable conditions) was never below 140°. The existence of stable and unstable hydrophobicity adds a new “piece” in the big picture of water-repellency development after surface texturing by laser pulses. Unfortunately, these measurements cannot offer an answer to the still opened and important question, what is the main reason (mechanism) for the superhydrophilic-to-superhydrophobic transition of the laser-textured surfaces. This question was addressed by several authors who proposed different (even contradictory) mechanisms, including partial surface deoxidation [43] and the creation of hydrophobic functional groups [33]; decomposition of carbon dioxide into carbon with active magnetite [25]; as well as absorption of organic matter from the atmosphere, where the processed samples are stored [44].

The existing literature [12,13,14,15,25,33,34,37] evaluated the wettability of the metallic surfaces after laser texturing only within a short-term period. As presented by our results in Figure 8, consideration of just the short-term contact angle development leads to the (wrong) conclusion that the “final” contact angle is strongly influenced by the scan line separation, since in a short-term period the smallest scan line separation (i.e., *Δx* = *Δy* = 10 μm) results in a (super)hydrophilic surface. A similar conclusion was reached by Ta et al. [13] who examined the surface wettability only within 15 days. However, already intermediate-term evaluation (i.e., after 3 months) reveals that such hydrophilic state is not stable, since hydrophobicity (*θ* > 90°) is achieved on all samples, independent of the scanning line separation. Nevertheless, to get the whole and complete insight into the hydrophilic-to-hydrophobic transition, the long-term evaluation of the contact angle development should be performed. In this case, one can observe that all the samples exhibited a successful transition from the superhydrophilic to the hydrophobic state, and that the superhydrophobic state (*θ* > 150° and RoA < 5°) is achieved even for the smallest scan line separation (i.e., *Δx* = *Δy* = 10 μm; Figure 8a).

The presented long-term measurements, in contrast to the existing literature [13], suggest that the highest final contact angle is achieved for highest values of the totally absorbed energy. In case of smaller scanning line separation, more pulses (i.e., more total energy) are needed to process the whole surface. Therefore, from the presented results, it can be concluded that a higher amount of the totally absorbed energy leads to a slower development of superhydrophobicity after laser texturing. Furthermore, the results in Figure 6 and Figure 8 indicate that this slower wettability transition leads to higher final contact angles (more hydrophobic surfaces).

The importance of the long-term wettability measurements is additionally proved by the examination of how the focal position influences on surface wettability (Figure 9). Here, the short-term measurements lead to the (wrong) conclusion that the processing out of the focus causes a more hydrophilic behavior. A similar conclusion was developed by Ta et al. [14] who demonstrated that wettability gradients can be achieved by processing the metallic sample at different focal positions; unfortunately, their measurements were limited to less than 2 months. Our long-term contact angle measurements reveal that such wettability gradients are not stable by time. Instead, the presented results clearly indicate that the focal position (within the range that still enable pulse fluences that are high enough for the laser ablation) mainly influences the wettability transition, but not the final contact angle. Additionally, these results prove that the slower the hydrophilic-to-hydrophobic transition is, the higher the final contact angle can be expected.

Not only the processing parameters, but also the measurements of the contact angle itself influence the wettability transition. This happens because the measurement always interferes with the measured result—in this case, it is impossible to measure the contact angle without wetting the surface by putting a droplet on it. The influence of the contact angle measurements on wettability behavior (Figure 10) indicates that frequent measurements speed up the wettability transition, but have no significant influence on the final wettability. Therefore, the same period of the contact angle measurements should be used, when one aims to compare the wettability transition of different surfaces. On the contrary, it seems that the measurement frequency is not very important when only the final contact angles (measured after steady-state conditions are achieved) are investigated and/or compared.

## 5. Conclusions

The as-received stainless-steel surfaces have been textured with nanosecond pulses using different pulse fluences and different scan line separations. The short- (within 40 days), intermediate- (within 100 days) and long-term (after one year) superhydrophilic-to-(super)hydrophobic transition was examined in the context of the following processing and environmental parameters: *(i)* pulse fluence, *(ii)* scan line separation, *(iii)* focal position and *(iv)* wetting period (due to the measurement of the contact angle). The presented results lead to the following conclusions:

- Depending on laser fluence, the laser-textured surfaces can develop stable or unstable hydrophobicity; in our case, the stable conditions were achieved if the peak fluence exceeded the threshold fluence of *F*_0_ = 12 J cm^−2^. In this case, all final contact angles were above 140°. If the fluence was below this threshold, the surface first became hydrophobic and after achieving the maximal contact angle, its hydrophobicity decreased by time.
- The short-term evaluation (e.g., within only 2 months) that is presented by the majority of papers covering this topic, can lead to wrong conclusions, such as stable hydrophilicity for smaller scan line separations or appearance of the wettability gradients due to processing at different focal positions. Here, a long-term examination reveals that such surfaces tend to become hydrophobic after a long-enough period.
- The presented results indicate that a faster development of hydrophobicity immediately after the laser texturing usually leads to a lower final contact angle and vice versa, if this transition is really slow (as in our case of 10-μm scan line separation), larger contact angles or even superhydrophobic surfaces exhibiting the self-cleaning effect are expected when the transition is over and the stable conditions are achieved.
- The wetting period due to the measurements of the contact angle influences the hydrophilic-to-hydrophobic transition, but it appears to have no influence on the final wettability (the final contact angle), when stable conditions are achieved.

## Figures and Tables

**Table 1:** Average surface roughness (*S*_a_) for surfaces from Figure 2.

*Δx*, *Δy* (μm)	*S*_a_ (μm)	
	*F*_0_*=* 12.1 J cm^−2^	*F*_0_*=* 25.1 J cm^−2^
10	0.85	1.47
25	1.13	1.70
50	4.54	5.40
100	1.71	4.02

## Supplementary Materials

The following are available online at http://www.mdpi.com/1996-1944/11/11/2240/s1, Figure S1: Determination of the static contact angle, Figure S2: High-magnification SEM of S68, Figure S3: High-magnification SEM of S18, Figure S4: High-magnification SEM of S32, Figure S5: Self-cleaning effect, Table S1: Parameters of laser texturing with different fluences, Table S2: Parameters of laser texturing with different scan line separations, Table S3: Parameters of laser texturing at different focal positions, Table S4: Parameters of laser texturing for determination of the threshold fluence for laser ablation.
