# Supplementary material for: Long-Term Influence of Laser-Processing Parameters on (Super)hydrophobicity Development and Stability of Stainless-Steel Surfaces
Source: Materials (Basel). 2018 Nov 11;11(11):2240. doi: 10.3390/ma11112240 (PMC6266256; doi:10.3390/ma11112240)
Supplement: Supplementary file 1 [file materials-11-02240-s001.pdf]

Supporting Information

Long-term influence of laser-processing parameters on (super)hydrophobicity development and stability of stainless-steel surfaces

Peter Gregorčič[1]*, Marjetka Conradi[2], Luka Hribar[1] and Matej Hočevar[2]

[1] Faculty of Mechanical Engineering, University of Ljubljana, Aškerčeva 6, 1000 Ljubljana, Slovenia
[2] Institute of metals and technology, Lepi pot 11, 1000 Ljubljana, Slovenia
* Correspondence: peter.gregorcic@fs.uni-lj.si; Tel.: +386-1-477-1-172

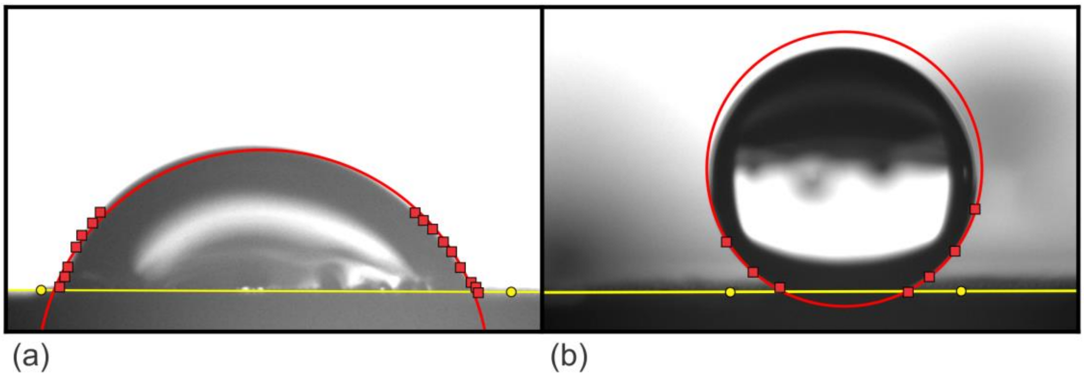

Figure S1. Determination of the static contact angle

The static contact angle was determined as shown in Figure S1. We fitted the line (in yellow) and the circle (in red) to the control points. The control points for the line are shown by the yellow circles and were manually added into the solid-gas interface. The control points for the liquid-gas interface (the red squares) were also manually added in such a way that the fitted circle corresponds to the liquid-gas interface in the vicinity of the contact points among all three phases: gas, liquid and solid. Two examples are shown in Figure S1: (a) fitting the apparent contact angle on an asymmetrical droplet (θ = 68°); and (b) fitting the apparent contact angle in the case of a symmetrical droplet (θ = 153°).

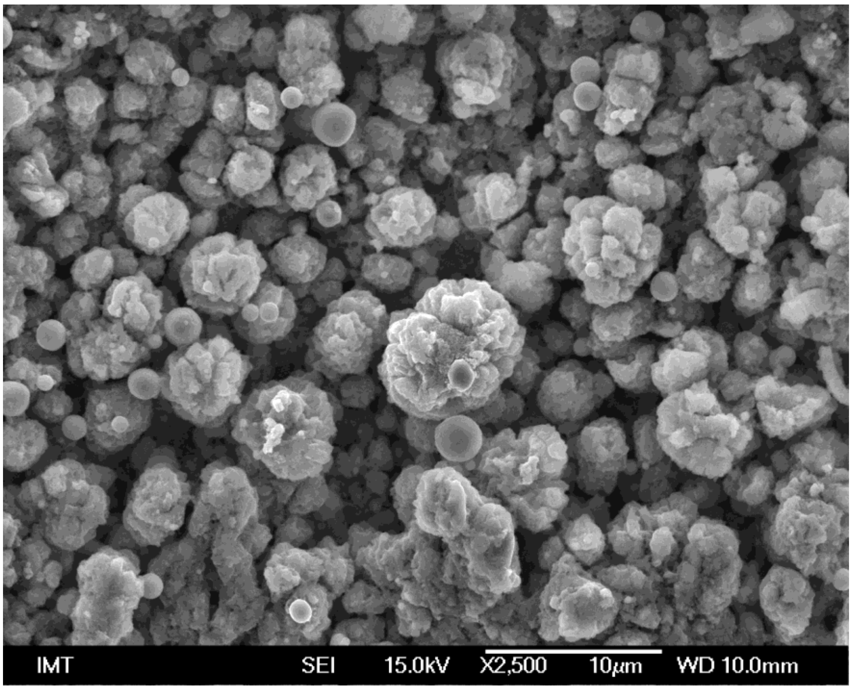

Figure S2. High-magnification SEM of S68

High-magnification image of the surface, processed by F_0 = 25.1 J/cm^2, at Δx = Δy = 10 µm (sample S68) is shown in Figure S2.

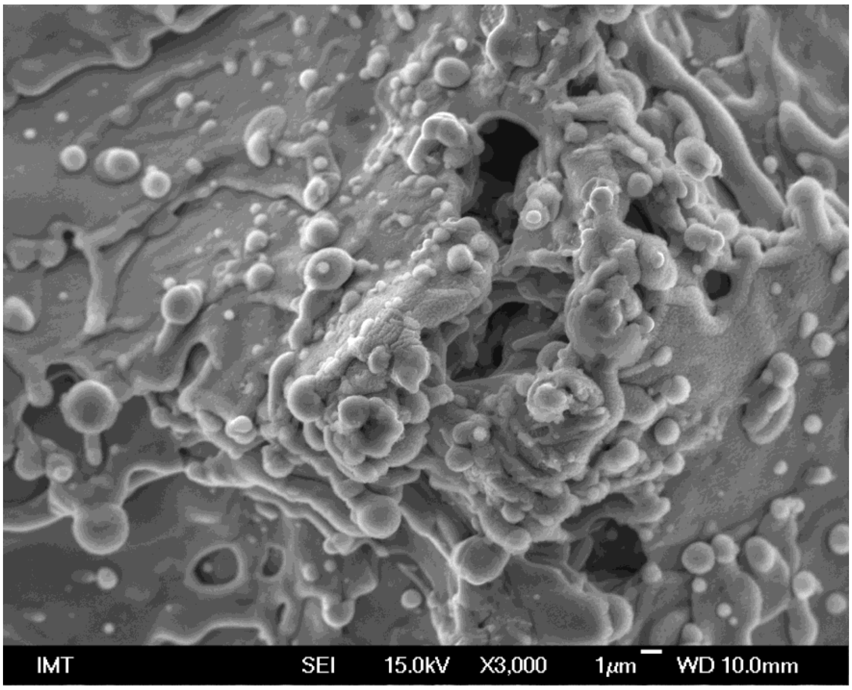

Figure S3. High-magnification SEM of S18

High-magnification SEM image of the surface, processed by F_0 = 12.1 J/cm^2, at Δx = Δy = 50 µm (sample S18) is shown in Figure S3.

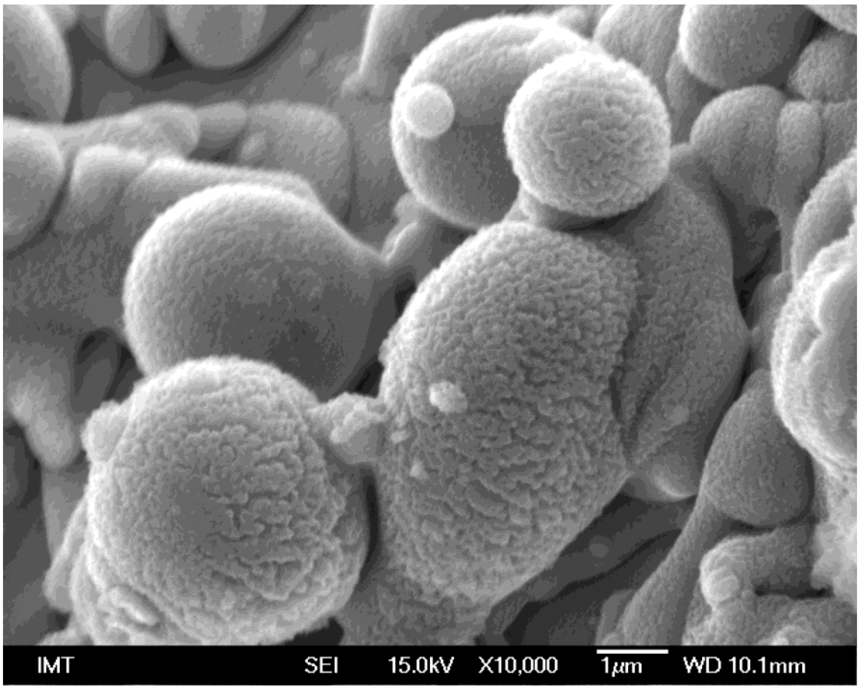

Figure S4. High-magnification SEM of S32.

High-magnification SEM image of the surface, processed by F_0 = 25.1 J/cm², at Δx = Δy = 50 μm (sample S32) is shown in Figure S4.

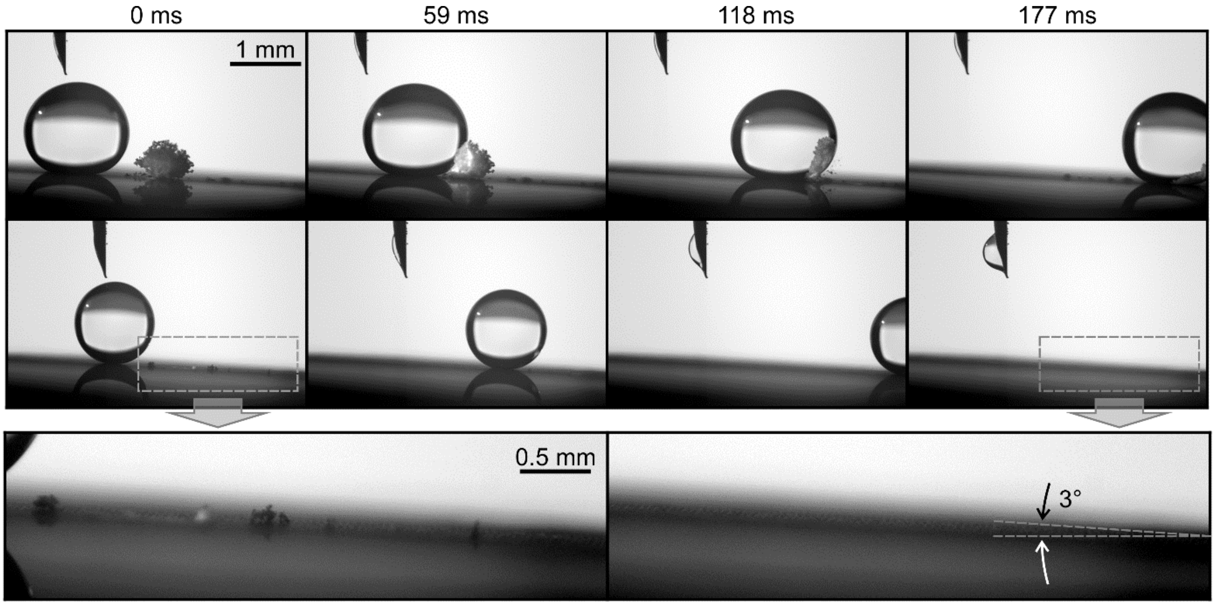

Figure S5. Self-cleaning effect

Self-cleaning effect on a laser-textured surface (F_0 = 25.1 J/cm², Δx = Δy = 10 mm) after one year of exposure to the atmospheric air is shown in Figure S5. The relative time that each image is captured is shown on the top of each image.

Table S1: Parameters of laser texturing with different fluences

Sample label (texturing direction)	Average power [W]	Pulse energy [µJ]	Peak fluence F_0 [J cm^{-2}]
S1 (0°/90°), S33 (0°)	1.1	44	3.33
S2 (0°/90°), S34 (0°)	1.2	48	3.63
S3 (0°/90°), S35 (0°)	1.4	56	4.24
S4 (0°/90°), S36 (0°)	1.5	60	4.54
S5 (0°/90°), S37 (0°)	1.6	64	4.84
S6 (0°/90°), S38 (0°)	1.8	72	5.45
S7 (0°/90°), S39 (0°)	1.9	76	5.75
S8 (0°/90°), S40 (0°)	2.1	84	6.36
S9 (0°/90°), S41 (0°)	2.2	88	6.66
S10 (0°/90°), S42 (0°)	2.4	96	7.27
S11 (0°/90°), S43 (0°)	2.6	104	7.87
S12 (0°/90°), S44 (0°)	2.9	116	8.78
S13 (0°/90°), S45 (0°)	3.1	124	9.39
S14 (0°/90°), S46 (0°)	3.3	132	9.99
S15 (0°/90°), S47 (0°)	3.5	140	10.6
S16 (0°/90°), S48 (0°)	3.8	152	11.5
S17 (0°/90°), S49 (0°)	3.9	156	11.8
S18 (0°/90°), S50 (0°)	4.0	160	12.1
S19 (0°/90°), S51 (0°)	4.7	188	14.2
S20 (0°/90°), S52 (0°)	5.0	200	15.1
S21 (0°/90°), S53 (0°)	5.2	208	15.7
S22 (0°/90°), S54 (0°)	5.7	228	17.3
S23 (0°/90°), S55 (0°)	6.0	240	18.2
S24 (0°/90°), S56 (0°)	6.4	256	19.4
S25 (0°/90°), S57 (0°)	6.8	272	20.6
S26 (0°/90°), S58 (0°)	7.0	280	21.2
S27 (0°/90°), S59 (0°)	7.1	284	21.5
S28 (0°/90°), S60 (0°)	7.4	296	22.4
S29 (0°/90°), S61 (0°)	7.5	300	22.7
S30 (0°/90°), S62 (0°)	7.8	312	23.6
S31 (0°/90°), S63 (0°)	8.1	324	24.5
S32 (0°/90°), S64 (0°)	8.3	332	25.1

Table S2: Parameters of laser texturing with different scan line separations

Parameter / Sample	Scan line separation	Average power [W]	Pulse energy [μJ]	Peak fluence F_0 [J cm^{-2}]
S65	0°; Δx = 10 μm	4.0	148	12.1
S66		8.3	332	25.1
S67	0°/90°; $\Delta x = \Delta y$ = 10 μm	4.0	148	12.1
S68		8.3	332	25.1
S69	0°; Δx = 25 μm	4.0	148	12.1
S70		8.3	332	25.1
S71	0°/90°; $\Delta x = \Delta y$ = 25 μm	4.0	148	12.1
S72		8.3	332	25.1
S73	0°; Δx = 100 μm	4.0	148	12.1
S74		8.3	332	25.1
S75	0°/90°; $\Delta x = \Delta y$ = 100 μm	4.0	148	12.1
S76		8.3	332	25.1
S77	0°; Δx = 200 μm	4.0	148	12.1
S78		8.3	332	25.1
S79	0°/90°; $\Delta x = \Delta y$ = 200 μm	4.0	148	12.1
S80		8.3	332	25.1

Table S3: Parameters of laser texturing at different focal positions

Parameter / Sample	Focal position Δz [μm]	Scan line separation	Average power [W]	Pulse energy [μJ]	Pulse fluence F [J cm^{-2}]
S81, S83-S86	0	0°/90°; $\Delta x = \Delta y$ = 50 μm	4.0	156	12.1
S82	-600				

The parameters of laser texturing at different focal positions and for testing wetting behavior as a function of the wetting period are given in Table S3.

Table S4: Parameters of laser texturing for determination of the threshold fluence for laser ablation

Scan line separation Δx [μm]	Average power [W]	Pulse energy [μJ]	Peak fluence F_0 [J cm^{-2}]	μ-channel diameter D_μ [μm]
50	1.49	60	4.51	16
50	2.24	90	6.78	32
100	3.7	148	11.2	42
50	3.95	158	12.0	43
100	8.3	332	25.1	57

The parameters of laser texturing (0°) at different fluences for surfaces that were evaluated for the determination of the threshold fluence for laser ablation are given in Table S4. The diameters of the μ-channels D_μ, measured from SEM images are also listed.
